# Relations of the Nervous System to Diseases of the Skin

**Published:** 1875-10

**Authors:** 


The Relations of the Nervous System to Diseases of the Skin. By L. Duncan Bulkley, A.M., M.D. New York: Geo. P. Putnam's Sons. Pamphlet; pp. 45.

